# Prevalence of *MEFV* gene mutations in apparently healthy Slovenian and Macedonian population

**DOI:** 10.1186/1546-0096-9-S1-P301

**Published:** 2011-09-14

**Authors:** M Debeljak, N Abazi, N Toplak, K Stavrić, M Kolnik, D Kuzmanovska, T Avčin

**Affiliations:** 1 Center for Medical Genetics, University Children's Hospital, University Medical Center, Ljubljana, Slovenia; 2 Department of Allergology, Rheumatology and Clinical Immunology, University Children's Hospital, University Medical Center, Ljubljana, Slovenia; 3 University Children Hospital, Medical Faculty, Ss. Cyril and Methodius University, Skopje, Republic of Macedonia

## Background

Familial Mediterranean Fever (FMF) is an autosomal-recessive disorder characterized by recurrent attacks of fever and serositis. It is common in eastern Mediterranean population. There are only few FMF patients in Slovenia and Macedonia and the mutation carrier rate is not known. So far, over 80 disease associated mutations have been identified in *MEFV* gene; the most common are M694V, V726A, M680I, E148Q and M694I.The distribution pattern of *MEFV* mutation along the Mediterranean Sea is not uniform; eastern populations have the highest number of carriers (20-39%), whereas the number of carriers in western Mediterranean populations is considerably lower.

## Aim

The aim of this study was to determine the carrier rate in apparently healthy Macedonian and Slovenian populations.

## Methods

We screened 100 subjects from both populations. Exon 10 was PCR amplified and screening was performed with dHPLC. All amplicons with detected nucleotide changes were subsequently sequenced with ABI Prism 310 Genetic analyzer. Amplicons of exon 2 were directly sequenced.

## Results

Heterozygous mutations were found in 7% of apparently healthy Slovenians and in 16% of apparently healthy Macedonians. Mutations found in Slovenian population were as follows: V726A (4), K695R (2) and E148Q (1). Mutations found in Macedonian population were as follows: E148Q (8), K695R (7) and M694V (1).

## Conclusion

We found higher than expected carrier rate in both populations, 7% and 16%, respectively. It is interesting to note that almost half of detected carriers in Macedonian and one third in Slovenian population have a K695R mutation.

